# When Watching Video, Many Saccades Are Curved and Deviate From a Velocity Profile Model

**DOI:** 10.3389/fnins.2018.00960

**Published:** 2019-01-07

**Authors:** Francisco M. Costela, Russell L. Woods

**Affiliations:** ^1^Schepens Eye Research Institute, Massachusetts Eye and Ear, Boston, MA, United States; ^2^Department of Ophthalmology, Harvard Medical School, Boston, MA, United States

**Keywords:** saccade, curvature, velocity profile deviation, gaze-contingent display, media

## Abstract

Commonly, saccades are thought to be ballistic eye movements, not modified during flight, with a straight path and a well-described velocity profile. However, they do not always follow a straight path and studies of saccade curvature have been reported previously. In a prior study, we developed a real-time, saccade-trajectory prediction algorithm to improve the updating of gaze-contingent displays and found that saccades with a curved path or that deviated from the expected velocity profile were not well fit by our saccade-prediction algorithm (velocity-profile deviation), and thus had larger updating errors than saccades that had a straight path and had a velocity profile that was fit well by the model. Further, we noticed that the curved saccades and saccades with high velocity-profile deviations were more common than we had expected when participants performed a natural-viewing task. Since those saccades caused larger display updating errors, we sought a better understanding of them. Here we examine factors that could affect curvature and velocity profile of saccades using a pool of 218,744 saccades from 71 participants watching “Hollywood” video clips. Those factors included characteristics of the participants (e.g., age), of the videos (importance of faces for following the story, genre), of the saccade (e.g., magnitude, direction), time during the session (e.g., fatigue) and presence and timing of scene cuts. While viewing the video clips, saccades were most likely horizontal or vertical over oblique. Measured curvature and velocity-profile deviation had continuous, skewed frequency distributions. We used mixed-effects regression models that included cubic terms and found a complex relationship between curvature, velocity-profile deviation and saccade duration (or magnitude). Curvature and velocity-profile deviation were related to some video-dependent features such as lighting, face presence, or nature and human figure content. Time during the session was a predictor for velocity profile deviations. Further, we found a relationship for saccades that were in flight at the time of a scene cut to have higher velocity-profile deviations and lower curvature in univariable models. Saccades characteristics vary with a variety of factors, which suggests complex interactions between oculomotor control and scene content that could be explored further.

## Introduction

It seems a common misperception that saccades are ballistic eye movements, displacing in straight lines, not modified during flight, and with a well-described velocity profile. In fact, plenty of recent research has investigated the deviations of saccades from a straight line, saccade curvature. In a previous study (Han et al., 2013), as a real-time, saccade-trajectory prediction algorithm was developed, it was noted that some saccades caused the algorithm to have greater errors. Those saccades did not fit the common perception that saccades are ballistic eye movements, not modified during flight, with a straight path and a well-described velocity profile. The saccade prediction algorithm was developed for use in a gaze-contingent visual display system. All systems have an updating latency of at least 10 ms (Santini et al., 2007) to 12 ms (Saunders et al., 2014) and most systems have a considerably longer updating latency (Saunders et al., 2014). That latency can become a problem when the viewer is making saccades, as the eye is no longer at the measured location by the time the display is updated, and thus there is an updating error. When implemented in a gaze-contingent system, the Han et al. (2013) saccade prediction did improve the gaze-contingent updating, but saccades with a curved path and or that had a velocity profile that was not typical (and thus not well fit with that model or other saccade models) had larger updating errors.

Gaze-contingent systems have been used in a variety of settings. Perhaps the settings in which gaze-contingent systems are most likely to be widely used are in real-world settings. This includes control or interaction based on eye movements or modifying the view during the conduct of activities of daily living. Most studies of saccades have been conducted in experimental settings with very constrained conditions such as moving fixation from one defined location to another defined location, often repeatedly, and usually without other stimuli present and a blank background. However, the real world is much more complex, so it is not clear that all that we have learnt from such studies applies when viewing natural stimuli, such as while walking (scene change and object motion created by self-motion) or viewing video, when there are complex scenes with objects moving within the scene. For example, Blackmon et al. (1999) tested very simple, synthetic dynamic scenes and reported some evidence for certain aspects of the “scanpath theory” (Noton and Stark, 1971). Modeling saliency, i.e., the contribution of low-level features to gaze control, was also important in a number of studies (e.g., Itti, 2005; Le Meur et al., 2007) that found that temporal change and motion predict eye movements strongly. As reported by Dorr et al. (2010), there are significant differences in gaze patterns between natural static scenes and dynamic scenes. Eye movements on static images are initially driven by stimulus onset effects and later by subject-specific idiosyncrasies. Conversation scenes are another example in which classical models of visual attention dramatically fail to predict eye positions. Coutrot and Guyader (2014) showed that faces, and particularly talking faces, are the features that best explain the gazes recorded during viewing of conversation scenes. Other authors have also studied gaze behavior in real-world tasks, such as driving (Land and Lee, 1994), food preparation (Land and Hayhoe, 2001), and walking around indoors (Munn et al., 2008) and outdoors (Cristino and Baddeley, 2009). While eye movements, gaze behavior and scanpath are of interest, very few studies have looked at the characteristics of the saccades made during such behavior. Here, we are interested in saccades made during a naturalistic task, watching Hollywood movies, which have not been studied before.

The path of a saccade is not always straight (Lamasky, 1869). Yarbus (1967) showed curved saccades made while scanning a two dimensional shape. Sheliga et al. (1994) were the first to study these curved trajectories, showing that directing covert attention to a spatial location influences eye movement trajectories (Sheliga et al., 1994, 1995a,b). These findings and subsequent studies on the activation of the oculomotor system in relation to spatial attention allocation (Van der Stigchel and Theeuwes, 2007) concluded that the strength of saccade deviation is a measure of the amount of attention allocated to any particular location in space. Interestingly, saccade deviations are also observed for reflexive shifts of visual attention without the presentation of a peripheral cue (Nummenmaa et al., 2009).

The time course of exogenous and endogenous activation in the saccade trajectory planning can explain a variety of eye movement data, including endpoints, latencies, and trajectories of saccades (Godijn and Theeuwes, 2002). Arai and Keller (2005) presented a model of saccade generation that accounted for curved saccades found during visual search tasks through a proposed delayed parallel input from the cerebellum to the saccadic burst generators in addition to that provided by the distributed input by the superior colliculus. Schreiber et al. (2006) reported that the curved trajectory of a significant proportion of catch-up saccades in the double step-ramp paradigm could be explained by an asynchrony between position and motion signals in saccade programming.

As indicated in the extensive review by Van der Stigchel (2010), many investigations of the curvature of saccade trajectories have studied the effects of distractors, including visual, auditory, and somatosensory cues (Doyle and Walker, 2001, 2002). Curvature can be toward or away from the distractor, may increase when the target is more predictable (Walker et al., 2006), and the amount of curvature may not systematically vary with the number of distractors (Arai et al., 2004). The trajectory of an erroneous initial saccade to a distractor is curved toward the goal of the subsequent saccade, suggesting that both movements are processed concurrently (McPeek and Keller, 2001). Curvature back toward the saccade goal may be attributed to a feedback system, with a separate representation of the visual target location, that enables an on-line correction of the saccade during mid-flight (McSorley et al., 2004). All of these studies have used very constrained, non-natural settings.

Deviations away from an element are observed in situations in which top-down preparation can influence the target selection process (Van der Stigchel et al., 2006). The salience of a second visual goal can be simultaneously maintained in the superior colliculus (McPeek and Keller, 2001) as it is presumably involved in the competitive neural interactions underlying saccade target selection (McPeek et al., 2003). Saccade curvature can provide a measure of processes that control and influence our behavior, during reading (Inhoff et al., 2010) or attention allocation to locations over time (Van der Stigchel and Theeuwes, 2007; Van der Stigchel, 2010; van Zoest et al., 2012). The only one of those studies that used a natural setting and task was the measurement of curved saccades during reading by Inhoff et al. (2010), where they found that curvatures increased with increases in the size of forward-directed saccades, regressions, and return sweeps. We are not aware of any studies that have investigated saccade curvature while viewing a dynamic scene.

Saccade velocity profiles are usually symmetrical at least for small and medium size saccades. An irregular velocity profile occurs when there is not a smooth acceleration or (more commonly) deceleration and may be determined by comparison to a typical saccade or the fit for a typical saccade (e.g., from a model). Overshoot, undershoot and glissades, which occur at the end of saccades, will not fit most models of saccades, and, consequently, display a velocity-profile mismatch (Bahill et al., 1975a). There are few reports of irregular velocity profiles, although they have been shown in eye gaze traces from monkeys (Figure 1 in Straube et al., 1997) and in a patient with Stiff-Person Syndrome with cerebellar degeneration (Zivotofsky et al., 2006).

Since we are using a gaze-contingent system during studies in which participants perform a natural-viewing task (watching videos), we wanted to explore the prevalence of these difficult-to-predict saccades and the circumstances in which they occur, or are more likely to occur. This would allow us to design studies that would reduce the prevalence of such saccades and, possibly, provide information that would increase our knowledge about the control and generation of saccades. To our knowledge, no prior studies have examined the patterns of saccadic curvatures or velocity-profile deviations while performing a natural-viewing task such as watching TV or movies. We suspected that the dynamic nature of video may elicit a high frequency of the difficult-to-predict saccades due to changing of visual elements on-screen during a saccade. Such changes, whether from action within the scene or from a scene cut, could act as attractor or repellor, by triggering adjustments from the oculomotor control system. This happens with hand and arm movements (i.e., if target is moved, the path of the hand will alter during its travel to the target; Bédard and Sanes, 2009). We hypothesized that the visual transitions introduced by scene cuts will serve as distractors, thus generating saccades with curvature and, perhaps, irregular velocity profiles (due to mid-flight alterations in the saccade path).

Further, we wanted to explore whether curvature and irregular-velocity profiles may vary between individuals (subject dependent), might vary according to characteristics of video content (video dependent), and might vary with characteristics of the saccade (saccade related, such as saccade direction) and might vary with time during the experimental session (e.g., fatigue). Thus, we conducted an observational study with hypotheses that examined certain features of the participant, eye movement and viewed material, to provide a first look at the prevalence and characteristics of saccade curvature and irregular-velocity profiles while watching videos.

## Materials and Methods

### Participants

Seventy-one normally-sighted participants (median age: 49.3 years) participated in two related experiments of a project that was approved by the Institutional Review Board of the Schepens Eye Research Institute in accordance with the Code of Ethics of the World Medical Association (Declaration of Helsinki). Informed consent was obtained both written and verbally from each participant prior to data collection.

Preliminary screening of the participants included self-report of ocular health, measures of visual acuity, and contrast sensitivity for a 2.5 degree-high letter target and evaluation of fixation and central retinal health using retinal photography (Nidek MP-1, Nidek Technologies, Vigonza, Italy or Optos OCT/SLO, Marlborough, MA, United States). All the participants had visual acuity of 20/25 or better, letter contrast sensitivity of 1.675 log units or better and steady central fixation with no evidence of retinal defects. For each participant we included gender, age, and education level (categorized within seven levels based on the International Standard Classification of Education, ISCED 2011) as demographic variables within our model. Education is a surrogate for socio-economic status which can be related to health status. The demographics of the participants are summarized in Table 1. Education information was not available for four of the 74 participants (22,984 saccades). Race-ethnicity information was not available for two participants.

**Table 1:** Self-reported demographic characteristics of participants.

**Gender**	Male	35 (49%)
	Female	36 (51%)
**Age (median, min-max)**		49.3 year (22–85 year)
**Race/Ethnicity**	Asian	3 (4%)
	Black	5 (7%)
	Hispanic	2 (3%)
	White	59 (83%)
**Highest education**	High school diploma	4 (6%)
	Some college	6 (9%)
	Associate degree	2 (3%)
	Bachelor's degree	25 (35%)
	Master's degree	17 (24%)
	Professional degree	5 (7%)
	Doctoral degree	8 (11%)

### Studies

We tracked the gaze of each subject as they viewed a 27” display (60 × 34 cm) from 1 m for a 33 × 19° potential viewing area with a table-mounted, video-based EyeLink 1,000 system (SR Research Ltd., Mississauga, Ontario, Canada). Subjects' head movements were restrained for the duration of the experiment using an SR Research head and chin rest. The clips were displayed and data collected with a MATLAB program using the Psychophysics (Brainard, 1997), Video (Pelli, 1997), and EyeLink Toolboxes. At the beginning of the experiment, the eye tracker was calibrated using a nine-point calibration procedure. If the average deviation exceeded 0.5° during this step, the calibration was performed again. In the first study, participants watched 40 to 46 of 206 30-s “Hollywood” video clips. At the beginning of each trial, participants were instructed to watch the stimulus “normally, as you would watch television or a movie program at home.” At the end of each clip, the participant was asked to describe the contents of the clip (Saunders et al., 2013). These participants (N = 61) contributed a total of 108,043 saccades to the dataset. Certain outcomes from this study were previously published (Costela et al., 2017). In the second study, fourteen participants (one was also in the first study) watched at least two of five different 30-min movie clips (*Bambi, Inside Job, Juno, Kpax*, and *Flash of Genius*), with calibration repeated every 5 min, contributing 110,695 saccades. A total of 218,744 saccades were identified from the two studies.

### Video Clips

For the first study, two hundred and six Hollywood video clips were chosen to represent a range of genres and types of depicted activities. The genres included nature documentaries (e.g., *BBC's Deep Blue, The March of the Penguins*), cartoons (e.g., *Shrek, Mulan*), dramas (e.g., *Shakespeare in Love, Pay it Forward*), comedies (e.g., *Julie and Julia, Adventureland*) and action (e.g., *Batman Forever, Eastern Promises*). The clips were 30 s long and were selected from parts of the films that had relatively few scene cuts, which was reflected in the average number of cuts per minute in our clips being 9, as compared to approximately 12 per minute in contemporary films (Cutting et al., 2010). The clips included conversation, indoor and outdoor scenes, action sequences, and wordless scenes where the relevant content was primarily the facial expressions and body language of one or more actors. For the second study, the five 30-min clips were from movies with content similar to the 30-s clips.

We hypothesized that video content might affect saccades. For example, videos that contain more man-made objects might be more visually cluttered than natural environments, and thus have more distractors that might increase saccade curvature. In a study of image enhancement that measured preference, participants preferred less enhancement (mainly of local contrast) in video clips when human faces were important, but not when human figures, man-made objects or nature were important (Satgunam et al., 2013). “Importance” because objects that are important for comprehension are likely to be attended and to be saccade targets. To investigate this hypothesis, the video content of all 206 30-s clips and for each 30-s segment of the five 30-min clips was rated for eight categories: (1) human faces; (2) human figures; (3) man-made objects; (4) nature; (5) auditory information; (6) lighting; (7) environment type; and (8) movie genre. Three observers who did not participate in the studies in which gaze was tracked were asked to count the number of scene cuts and rate each clip based on the importance of the categories human faces, human figures, man-made objects, nature and auditory information for understanding of the video content. Each rating scale ranged from 0 to 5, with 0 being absent and 5 being always present (or important), except for environment type that was binary (outdoor/indoor). To include video content in our model, video clips were considered to have high content (e.g., presence of faces was important) if the average rating was 4 or greater, and to have low content if the average rating was 1 or less, otherwise it was medium (rated 2 or 3). Time-on-task, or the time of occurrence of the saccade during the viewing session (order in intervals of 30 s) ranged from 1 to 60. Finally, to evaluate the effect of movie genre, clips were classified as dramas (*N* = 60), comedies (*N* = 49), action movies (*N* = 22), documentaries (*N* = 42), or animation (*N* = 42).

### Scene Cut (Shot Change) Detection

With a scene cut (to a new scene or a different viewpoint of the same scene) there is a sudden, unexpected change in the visual stimulus. For any saccade that was planned or in progress at the time of the scene cut, the scene changes during the saccade (sudden onset distractors) and the planned landing point no longer contains the object of interest (saccade target). Scene cuts in videos could act in the same way that distractors have been shown to influence in-flight saccade dynamics in research studies. To investigate this hypothesis, we detected scene cuts and compared those saccades that occurred over the scene cut to other saccades.

We implemented a version of the shot transition detection (SAD) method. Frames within each clip were converted to grayscale and we calculated the log-between-frame-pointwise difference in pixels, after normalizing by the size of the clip. We used a threshold based on the average of this metric plus one standard deviation to discriminate transitions that corresponded to scene cuts. This threshold returned a better sensitivity (0.7) than using two (0.55) or more standard deviations when compared with the number of cuts reported by the observers who counted scene cuts in 200 30-s clips.

For 58 clips, where the number of scene cuts calculated by our algorithm was lower than the reported number of cuts by observers, we added the timesample of the missing cut scenes by manually tracking the clips with QuickTime Player 7. The algorithm found some additional events in 43 clips. Some of these related to fast transitions (i.e., revolving doors or fast-motion panning) that we kept, as these would be expected to act as dynamic distractors. We removed those few cases where transitions were due to slow fading between scenes. This algorithm to detect scene cuts was applied to all 30-s and 30-min clips. In total, we identified 941 saccades (0.4%) that were in transition when a scene cut occurred.

Next, we examined the profiles of in-flight saccades, where the scene cut onset occurred within the saccade timesample, as compared with saccades happening away from the scene cut onset (i.e., saccades finished before the cut scene onset or triggered later in time).

### Saccade Detection

Many studies of saccades used non-natural viewing conditions in which there are few stimuli, the saccade target is stationary and easily identified and the interval between saccades is controlled and “long” (in the order of seconds), or natural tasks such as reading in which the stimuli are stationary and the background is simple (e.g., plain). In videos, saccade targets may not be obvious or unique, they may be moving (relative to the viewer), the inter-saccadic interval is often short (<200 ms; Otero-Millan et al., 2008), there may be many other objects within the scene creating visual clutter, and those may also be moving. The visual scene for many other natural tasks such as visual search, walking and driving are similar in their visual complexity. While watching videos, saccades may be preceded by a fixation or a pursuit and may end with a fixation or a pursuit, and that might vary depending on the conditions (e.g., orientation of saccade). Thus, detecting saccade initiation and completion is more difficult when using data from watching videos (and other natural viewing tasks). The most common method of saccade detection employs velocity or acceleration thresholds. Besides our own saccade detection algorithm (see details below), we employed an alternative algorithm, the Eye-Markup analysis tool (Berg et al., 2009), which provided comparable results (see Supplementary Material), although was found to be less efficient than ours by examining the main sequence of the detected saccades. We were unable to get access to the LNS algorithm (described as one of the most robust in a recent review on saccade detection algorithms Andersson et al., 2017). For a visual search task (static images), Dandekar et al. (2012) employed peak velocities and the acceleration window to identify saccade start and end. Konig and Buffalo (2014) used a novel method based on k-means cluster analysis to analyze visual search data (static images). We employed velocity criteria and additional criteria to remove eye-position overshoots and handle saccades that did not initiate or end with a fixation (including glissades).

**Figure 1:**
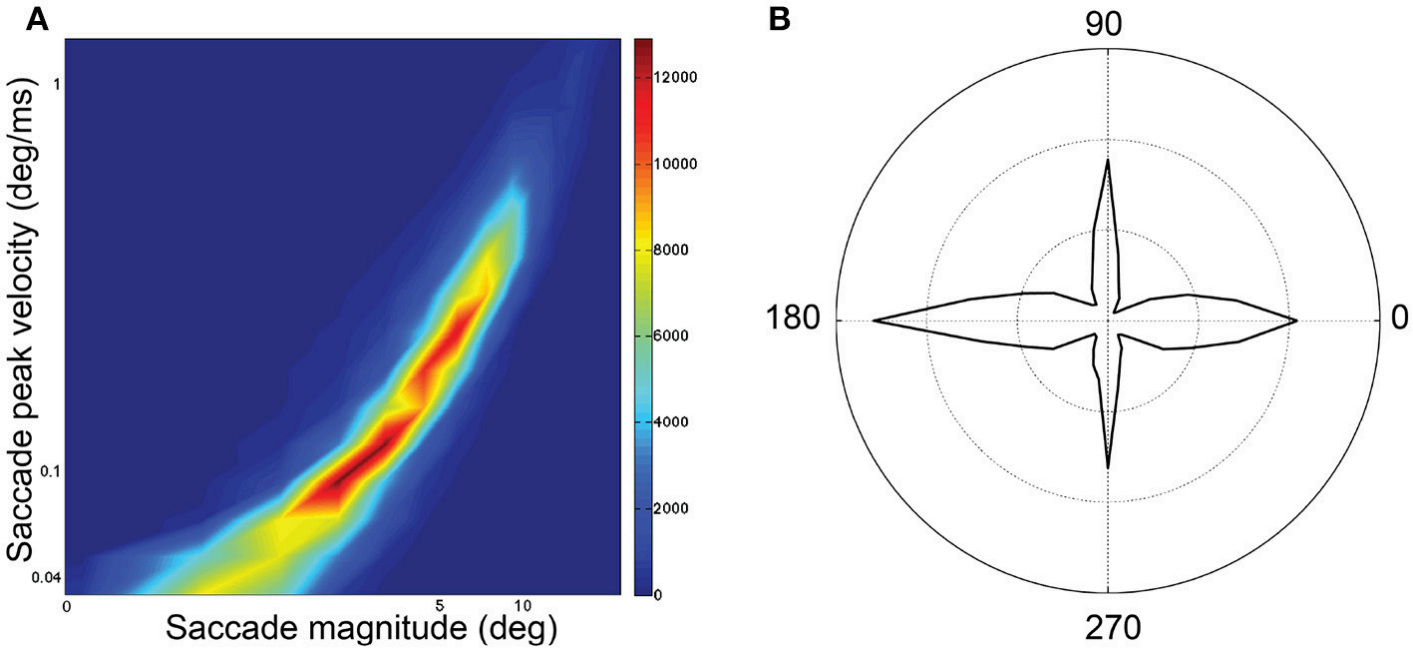
Saccadic features for all 74 subjects and 218,744 saccades. **(A)** Saccadic peak velocity–magnitude main sequence. The distribution is plotted on logarithmic scale where peak velocity is indicated on the y-axis and magnitude indicated on the x-axis. **(B)** Polar frequency distribution of saccade directions in polar co-ordinate orientations.

Sampling frequency was 1 kHz for all examples. Blinks were identified and removed using Eyelink's online data parser. Periods preceding and following these missing data were removed if they exceeded a speed threshold of 30°/s. Then, we interpolated over the removed blink data by applying cubic splines. For saccade detection in both data sets, the raw data was smoothed by applying a 3rd-order Savinsky-Sgolay filter with a frame size of 15. Without this smoothing, saccade detection was much less reliable. Speed was calculated as the norm of the velocity vector (speed), which is the first derivative of the eye position with respect to time. The beginning of a saccade was signaled when speed exceeded 30°/s for at least 10 ms. The end of a saccade was signaled when speed went below 30°/s. For analysis, the saccades were restricted to saccades (1) smaller than 40° as this was approximately the maximum diagonal dimension of the display; and (2) larger than 1° and 15 ms in duration to exclude microsaccades. We imposed additional restrictions regarding the initial (< 0.075°/ms) and terminal velocity (<0.3°/ms), as well as the removal of saccades with a velocity at first quartile of duration lower than 0.15 peak velocity. This threshold removed those eye movements with uniform but unrealistic low velocity profiles during their initial phase, and which may have been pursuit eye movements. This pool of saccades followed the saccadic peak velocity-magnitude relationship (Bahill et al., 1975b; Figure 1A) with a skewed distribution of magnitudes (median 4.13°) and durations (median 31 ms; **Figure 4C**). The main-sequence in our data when plotted on bi-logarithmic axes (Figure 1A) was not as linear as expected. Hamel et al. (2013) also showed curved saccades that did not fit the main sequence. The main sequence has been reported to vary between individuals (Bollen et al., 1993; Bargary et al., 2017) and between sessions (Bollen et al., 1993) and with factors such as saccade orientation (van Beers, 2007), saccade start location (van Beers, 2007), age (Dowiasch et al., 2015) and mental workload (Di Stasi et al., 2011). For each saccade, based on start location and landing point, we categorized saccade orientation as: *Horizontal* if path was 0 ± 5° or 180 ± 5° (Duke Elder and Wybar, 1973); *Vertical* if path was 90 ± 22° or 270 ± 22°; otherwise, the saccade orientation was considered *Oblique*. Saccades were predominantly horizontal and vertical, with fewer saccades being oblique (Figure 1B). The frequency distribution of saccade magnitudes is shown in Figure **4C**.

### Curvature

There have been different methods in the literature to define saccade curvatures (Van der Stigchel, 2010). Ludwig and Gilchrist (2002) reviewed prior methods and proposed using the area under the curve of a quadratic polynomial fit. Their model measures curvature as the deviation of a point from a line. But in their approach, the line is drawn between the start and end points of the saccade and curvature is quantified by whichever third point is farthest away from the line. It treats the saccade as if it contains only three measured points. This will yield an accurate representation of curvature only when the saccade has a perfect quadratic form, i.e., when the second derivative (the actual point-wise curvature) is a constant. If the saccade is not a perfect arc (specifically, one that can be fit with a quadratic) their method will not represent its actual curvature but the maximum deviation under the assumption that curvature is constant. The second derivative method is a standard mathematical measure of curvature (Coolidge, 1952; Casey, 2012) and is essentially equivalent to the method of Ludwig and Gilchrist (2002) when curvature is constant. However, when saccade curvature varies along the saccade, the second-derivative measure will vary from that found using a quadratic polynomial fit. When comparing curvature measures, Van der Stigchel et al. (2006) stated that “conclusions usually do not hinge on the exact measure chosen.” Here we used the median pointwise curvature as our saccade curvature metric, which it is based on the second derivative method, and was reported to be a reliable measure by Flynn and Jain (1989).

We defined curvature, *k*, as the median pointwise curvature, *k*_*t*_:

(1) $$\begin{array}{l} k_{t}=\frac{\sqrt{{\left(x_{t+1}-2x_{t}+x_{t-1}\right)}^{2}+{\left(y_{t+1}-2y_{t}+y_{t-1}\right)}^{2}}}{\sum_{t}\sqrt{{\left(x_{t}-x_{t-1}\right)}^{2}+{\left(y_{t}-y_{t-1}\right)}^{2}}}\\ \;t=\left\{2,3,...,T-2,T-1\right\} \end{array}$$

(2) $$k_{t}\;=\frac{\sqrt{{\left(\Delta x_{t+1}-\Delta x_{t}\right)}^{2}+{\left(\Delta y_{t+1}-\Delta y_{t}\right)}^{2}}}{\sum_{t=2}^{T-1}\sqrt{\Delta x^{2}+\Delta y^{2}}}$$

**Figure 2:**
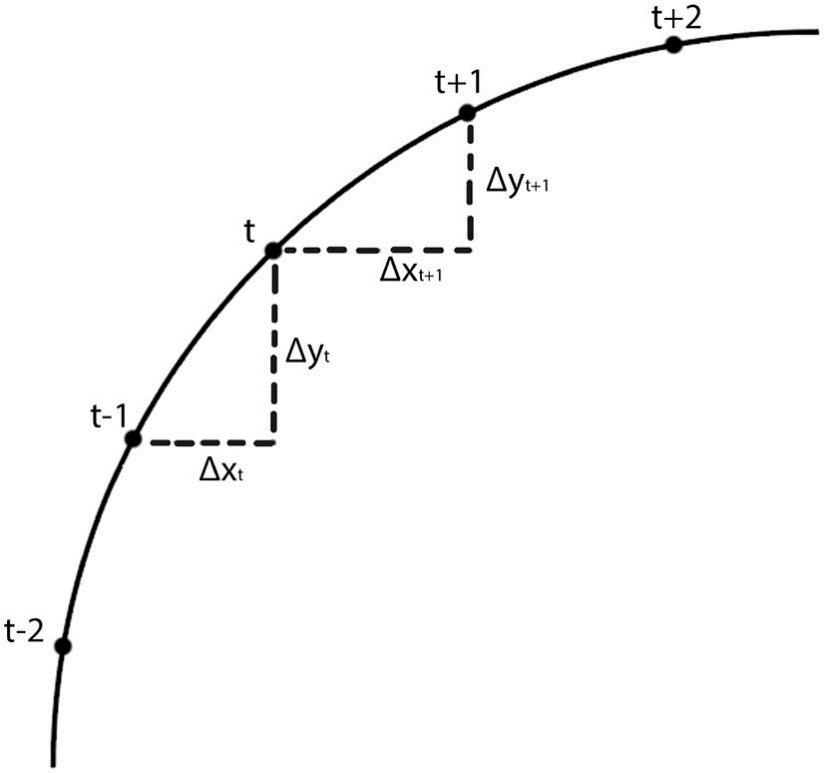
Schematic illustration of the calculation of saccade curvature. As detailed in Equation 1, saccade curvature compares the change in the vertical and horizontal dimensions between the change at sample *t* with the change at sample *t* + 1.

**Figure 3:**
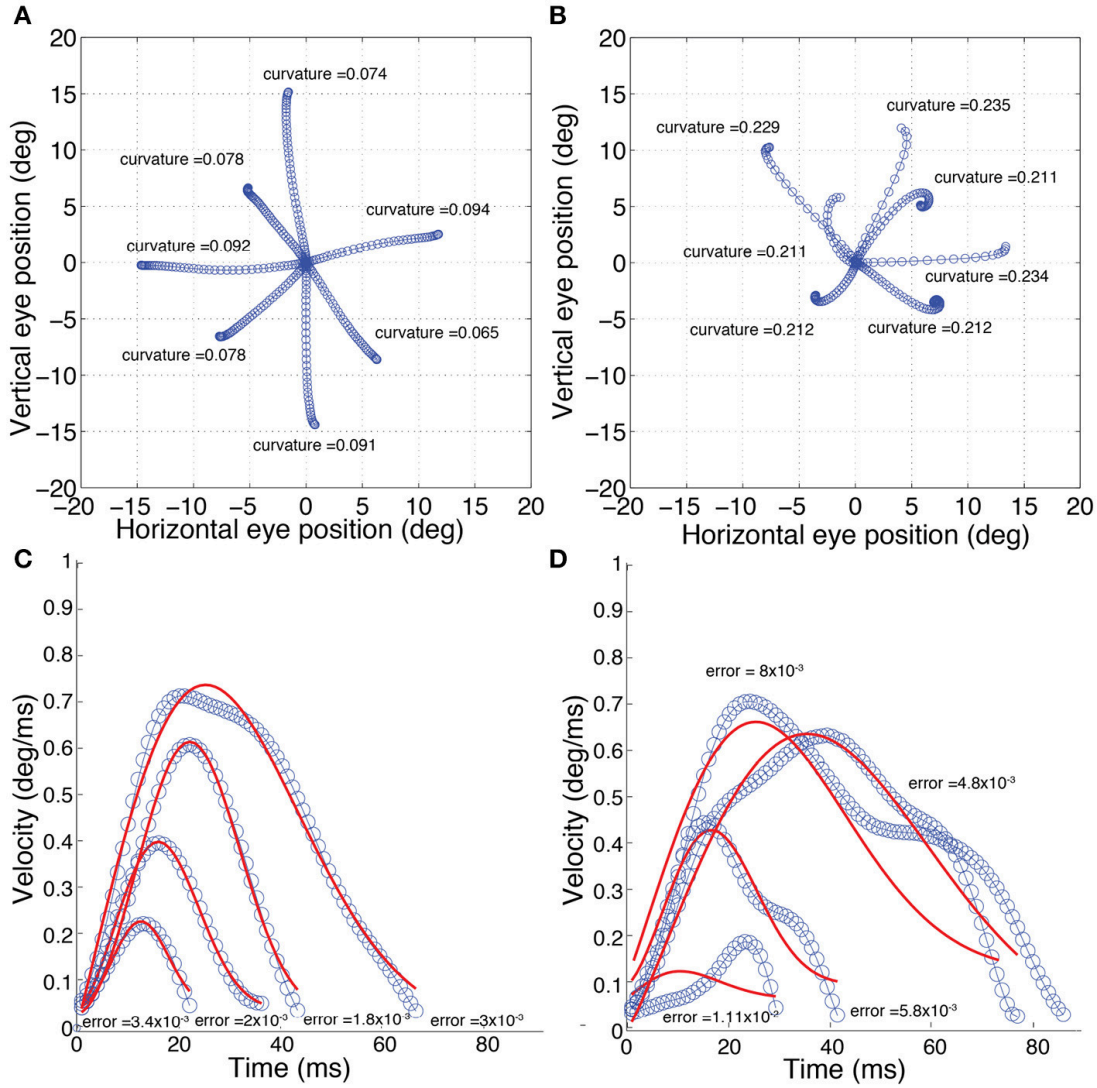
Examples of normal (left) and difficult-to-predict (right) saccades (as defined in the Methods section). **(A)** Saccades with “low” curvature; *k* < 0.21. **(B)** Saccades with “high” curvature; *k* > 0.21. **(C)** Saccades with “low” velocity-profile deviation; *velocity-profile deviation* < 4 × 10^−3^. **(D)** Saccades with “high” velocity-profile deviation; *velocity-profile deviation* > 4 × 10^−3^. The solid red lines in **(C,D)** show the fit using a compressed exponential model (Han et al., 2013).

**Figure 4:**
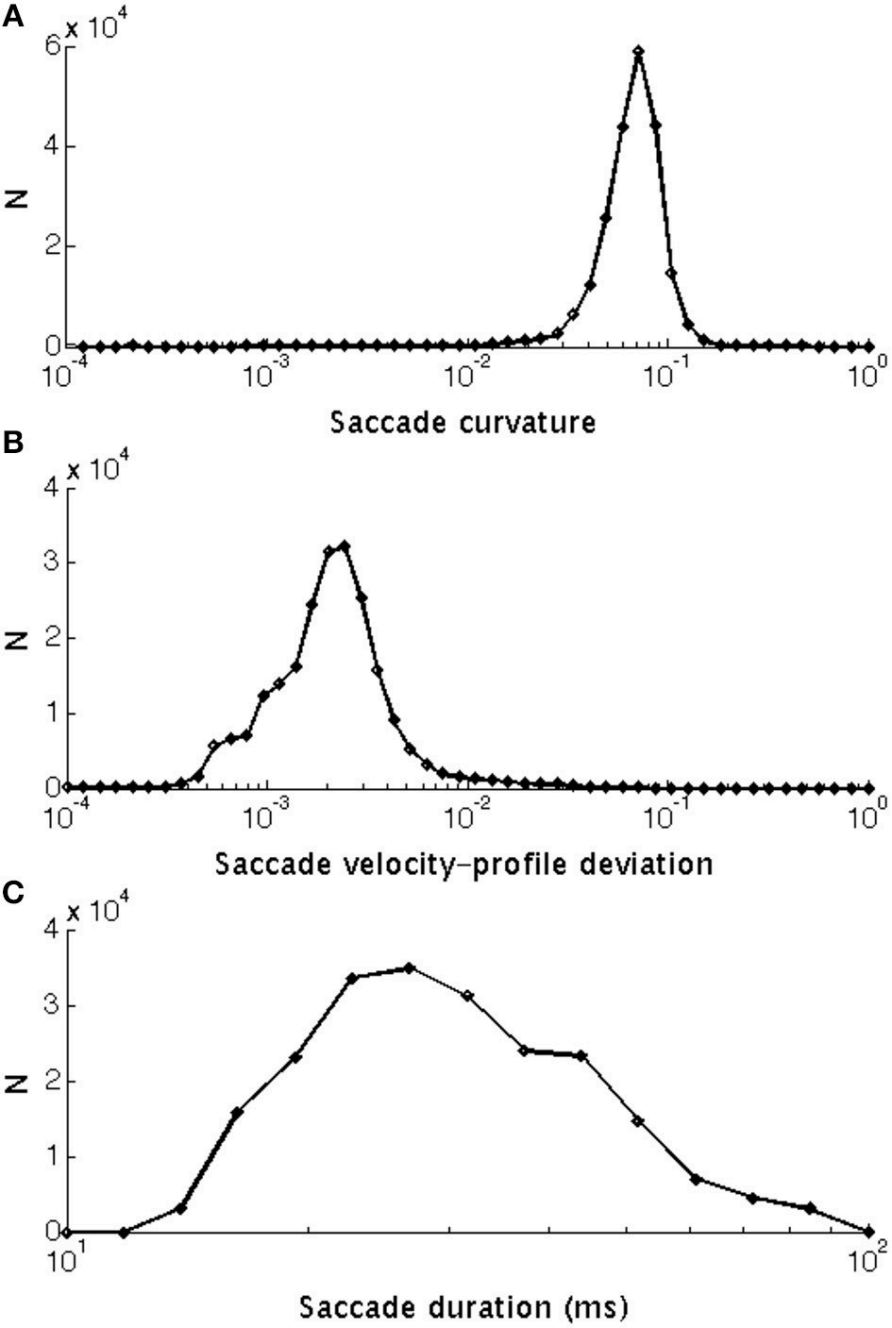
Frequency histograms of: **(A)** Saccade curvature that ranged from 0.0023 to 0.563 units (median: 0.075 units). **(B)** Saccade velocity-profile deviation that ranged between 4.87 × 10–5 and 0.078°/ms (median: 0.0023°/ms). **(C)** Saccade duration that ranged between 16 and 99° (median: 31°). All are shown with a log_10_ scale and the bins have been equally spaced logarithmically as the raw distributions were skewed.

The calculation of *k*_*t*_ is illustrated in Figure 2. If the line is straight, then *k*_*t*_ = 0. Saccades with low and high curvature are illustrated in Figures 3A,B, respectively. The frequency distribution of saccade curvature is shown in Figure 4A.

### Velocity-Profile Deviation

Earlier studies have noted the skewness in the shape of the velocity profile (Baloh et al., 1975; van Opstal and van Gisbergen, 1987). Baloh et al. (1975) fitted an exponential curve to magnitude-velocity plots to find that skewness increased with magnitude. van Opstal and van Gisbergen (1987) proposed five different measures of the degree of asymmetry in the saccadic velocity profile with the density function of the gamma distribution returning the best correlation between magnitude and skewness (*r* = 0.71). They also found a stronger correlation between saccade duration and skewness (*r* = 0.88), where saccades with large differences in magnitudes, but fixed duration, appear to keep the same skewness. Similarly, Takagi et al. (1993) found a close relationship between duration and skewness ratio (the proportion of acceleration time to total saccadic duration) where the ratios of temporal saccades were smaller than nasal ones. Chen et al. (2002) also applied a gamma function to the velocity profile to determine that at the same magnitude, skewness can be predicted from the peak velocity. We used a compressed exponential model, where one parameter in the generalized exponential model (*p*_3_ >1 in Equations 3, 4), accounts for the slower deceleration than acceleration phase (Han et al., 2013). We defined velocity-profile deviation as the mean-square error between the measured velocity and the best fit to the compressed exponential model.

For this study, we fit the saccadic eye movement trajectory with a compressed exponential model (Han et al., 2013):

(3) $$f\left(t\right)=p_{1}\left[1-\exp\left[-{\left(\frac{t}{p_{2}}\right)}^{p_{3}}\right]\right]$$

where p1, p2, and p3 are three parameters that need to be determined in data fitting, and t is the time since saccade onset. The parameter *p*1 corresponds to the amplitude of saccade. The parameter *p*2 can be used to rescale the saccade duration, and parameter *p*3 is used to control the decay trend This function is called the generalized exponential function because of the additional parameter *p*3. With *p*3 = 1, the generalized exponential function becomes the standard exponential function.

For our study, as deviations were most obvious in the velocity profile, though we fit in the position domain using Equation 3, we used errors in velocity instead of position. Given that the fit is the same, the errors would be linearly related to the fit in the position domain, so we would not expect any substantive difference in our subsequent analyses if we had used positional error. Saccades with low and high velocity-profile deviations are illustrated in Figures 3C,D, respectively

The velocity profile of the saccadic trajectory is the differential of Equation 3 (Han et al., 2013):

(4) $$f^{\prime }\left(t\right)=p_{1}p_{2}{}^{-1}p_{3}{\left(\frac{t}{p_{2}}\right)}^{p_{3}-1}\exp\left[-{\left(\frac{t}{p_{2}}\right)}^{p_{3}}\right]$$

Equation 4 is very similar to the probability density function of the Gamma distribution used by van Opstal and van Gisbergen (1987) to investigate the skewness of saccadic velocity profiles. We defined velocity-profile deviation as the root-mean-square error of the fit in Equation 4. See frequency distribution of velocity-profile deviations in Figure 4.

### Statistical Analyses

In a preliminary analysis, we did not find any substantive differences between the saccade data collected from the two studies (30-s clips vs. 30-min clips), so we merged the data into a single dataset and considered all of the available data without a term to differentiate between the two studies. For the analyses, the five 30-min clips were treated as 300 30-s segments.

**Figure 5:**
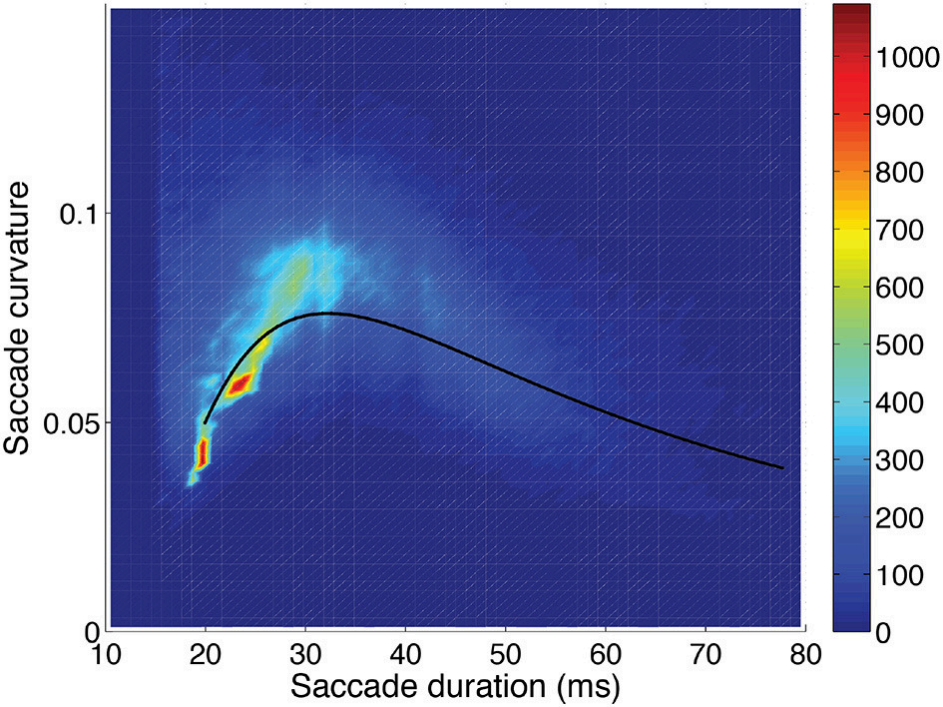
Saccadic curvature as a function of saccadic duration, showing the frequency scale on the right. A fit of the saccade curvatures (log_10_) using a cubic polynomial (uni-variable relationship, Table 2) is shown as the solid line.

**Figure 6:**
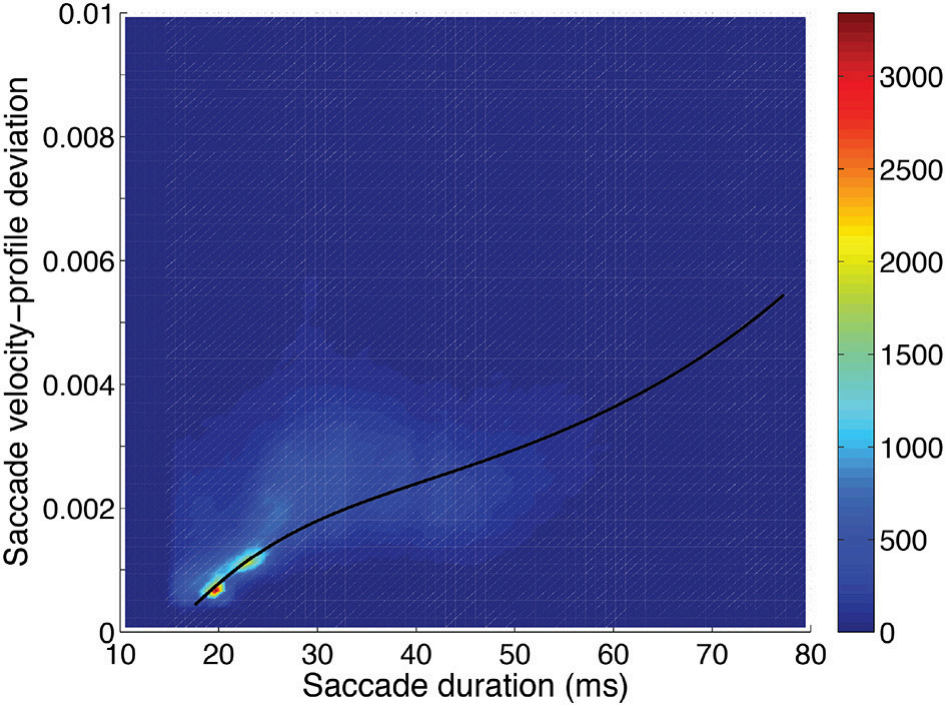
Saccadic velocity profile deviation as a function of saccadic duration showing the frequency scale on the right. A fit of the saccade velocity profile deviations (log_10_) using a cubic polynomial (uni-variable relationship, Table 2) is shown as the solid line.

**Figure 7:**
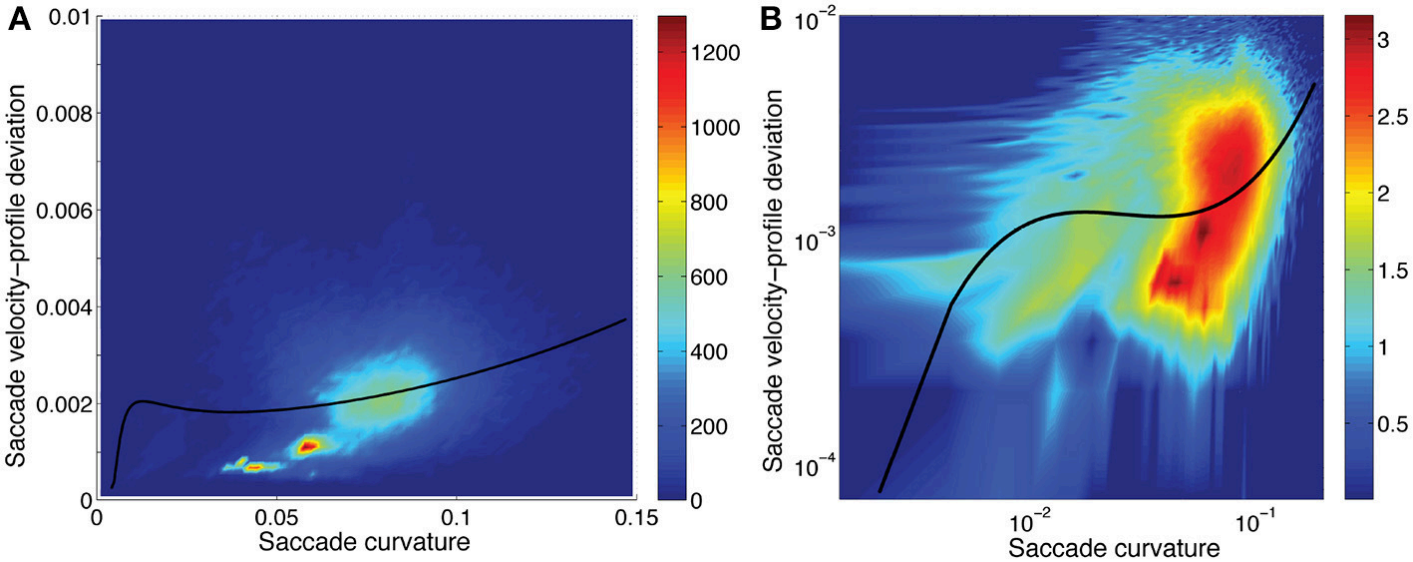
Saccadic velocity-profile deviation as a function of saccadic curvature showing the frequency (scale on the right). A fit of the saccade velocity-profile deviations (log_10_) using a cubic polynomial (uni-variable relationship, Table 2) is shown as the solid line. While the fit line does not pass through what appear to be the most-dense regions of data **(A)**, the residuals were well distributed, in the log-log domain of the fit **(B)**. Note that the distribution has a long tail to higher velocity-profile deviation values when plotted in the linear-linear domain, as here. **(B)** has the three axes in logarithmic domain.

The distributions of velocity-profile deviation, and saccade duration (or magnitude) were highly positively skewed, while curvature was mildly skewed, with all having a very small number of high values. Those distributions became approximately normal (Gaussian) when logarithmic values were used (frequency distributions are shown in Figure 4). For analyses, we used log_10_ values of curvature, velocity profile deviation, and saccade duration. The relationships between curvature, velocity profile deviation, and saccade duration (or magnitude) were non-linear. To examine the relationships, we investigated a variety of non-linear approaches including restricted cubic splines, and settled on the inclusion of square and cubic terms (of the log_10_ transformed values) for velocity profile deviation and saccade duration when predicting curvature and curvature and saccade duration when predicting velocity-profile deviation, as the residuals were not patterned. Figures 5–7 show the univariate relationships between curvature, velocity profile deviation, and saccade duration, when square and cubic terms were included (cubic polynomial fits in log_10_-log_10_).

We conducted crossed-random, mixed-effects regression analyses (Stata v14, College Station, TX) to determine which parameters had effects on the curvature and velocity-profile deviations. We present (1) “uni-variable” models in which a single predictor (independent) variable (fixed effect) was included in the model (forty models in total); and (2) “multi-variable” models in which all potential predictors were initially included and then predictors that were not significant (*p* > 0.01) were removed sequentially (one at a time) until all predictors in the model were significant (p ≤ 0.01; backwards regression method, two models in total). In the uni-variable models for factors curvature, velocity-profile error and duration, square and cubic terms were included in each model. As we had many models, we chose the *p* = 0.01 criterion to reduce the risk of false-positive findings (as compared to the commonly used criterion of *p* = 0.05). Backwards regression in the multi-variable models can remove variables that are highly correlated with another predictor (collinear), even when that predictor is significant in the uni-variable model. The fixed-effect factors were saccade-dependent (magnitude or duration, orientation); subject-dependent (age, gender, maximum education level); video-dependent (number of cuts, lighting level, environment, nature importance, auditory information presence, face importance, man-made object importance, human figure importance, movie genre); and time-dependent (time of saccade occurrence within the experimental session, time of in-flight saccade occurrence during scene cut). There were many strong correlations between the video-dependent factors, such as lighting level and man-made-object importance (r = -0.50) or human-figure importance (*r* = −0.45) and between man-made-object importance and human-figure importance (*r* = 0.72). In the multi-variable models, all factors should be independently predictive, and are corrected for the other predictors. There was no obvious multi-collinearity in the reported models.

**Table 2:** Summary of the regression coefficients of factors in “uni-variable” models of log_10_(curvature) and log_10_(velocity-profile deviation).

	**Log-curvature**			**Log-velocity-profile deviation**		
	**coefficient**	***z*-value**	**significance**	**coefficient**	***z*-value**	**significance**
Log-Curvature	−	-	−	3.15	29.5	<0.0001
Log-curvature^2^	−	-	−	1.76	27.7	<0.001
Log-curvature^3^	−	-	−	0.322	22.7	<0.001
Log-Velocity-profile deviation	2.00	29.3	<0.001	-	-	−
Log-deviation^2^	1.09	40.2	<0.001	-	-	−
Log-deviation^3^	0.180	49.8	<0.001	-	-	−
Log-Duration	13.4	56.5	<0.001	31.7	85.4	<0.0001
Log-duration^2^	−7.26	48.7	<0.001	−19.4	83.1	<0.001
Log-duration^3^	1.24	39.9	<0.001	4.05	83.2	<0.001
Horizontal orientation	−0.0215	25.9	<0.001	0.00757	4.96	<0.001
Vertical orientation	−0.00388	4.82	<0.001	0.0935	63.8	<0.001
Oblique orientation^*^	0.0169	25.1	<0.001	−0.0705	57.4	<0.001
Leftward saccade^*^	0.00475	3.27	0.001			0.40
Downward Saccade^*^	0.0367	27.7	<0.001	−0.0492	20.4	<0.001
Time-on-task			0.04	0.000162	3.90	<0.001
Inflight at scene cut	−0.0191	3.77	<0.001	0.0897	9.60	<0.001
Number of cuts			0.52			0.03
Environment (outdoor)			0.02			0.86
Nature-content importance			≥0.25	0.0153	3.68	<0.001
Face importance			≥0.06	−0.0125	3.28	0.001
Man-made importance			≥0.025	0.0145	2.66	0.008
Human figure importance	0.0187	3.68	<0.001	0.0345	5.75	<0.001
Lighting			0.04			0.04
Auditory information			≥0.57			≥0.48
Movie genre^∇^			≥0.28			0.02
Age	0.00037	4.83	<0.001			0.30
Gender	0.022	8.18	<0.001			0.64
Education			0.10			0.62

A positive coefficient indicates that the dependent variable (curvature or velocity-profile deviation) increased with an increase in the value of the variable or increased in the presence of that variable (for binary variables). (^*^) Horizontal saccades were compared to not-horizontal saccades and likewise for vertical and oblique saccades. Leftward saccades were compared to rightward saccades. Downward saccades are compared to upward saccades. (^∇^) Comedy had lower curvature and velocity-profile deviation compared to each of the other movie genres, documentary, animation, drama and action.

van Opstal and van Gisbergen (1987) found a stronger correlation of saccade duration with skewness than with saccade magnitude. In our sample, the correlation between saccade duration and saccade magnitude was *r* = 0.74. We report models of both curvature and velocity-profile deviations using the duration of the saccade instead of the magnitude. Models using saccade magnitude were similar to those found using duration (slightly higher Akaike information criterion). The Kolmogorov-Smirnov two-sample test was used to examine differences between distributions of saccades. This tests for both location and shape of the two distributions.

Participant and video clip were crossed-at-random factors in all models. For all models, random slopes for duration, and saccade curvature or velocity-profile error were included if the variable was a fixed effect rather than the dependent variable, for both participant and video clip. These random slopes were included as these were the most strongly-associated fixed factors (independent variables). Inclusion of random slopes for all variables was not possible with the computers available to us, as the models became too large and complex when including those additional 19 fixed factors in the full model. Inclusion of those random slopes improved the models over random intercepts only (Likelihood ratio test).

## Results

### Uni-Variable Relationships

Saccade curvature and velocity-profile deviation were related to each other, and both were related to saccade duration (or length) and orientation (Table 2). Figures 5–7 show the uni-variable relationships between saccade duration, saccade curvature and velocity-profile deviation. The relationships were non-linear. Cubic polynomial fits to the curvature and velocity-profile deviation were used within the crossed-random, mixed-effects regression models. As shown in Table 2, many of the potential predictor (fixed-factor) variables were related to saccade curvature and velocity-profile deviation in the uni-variable models. The strongest relationships with curvature and velocity-profile deviation were with saccade-dependent predictors (saccade duration and direction). There were some weaker relationships with time-dependent predictors (time within the session, in-flight saccade during scene cut), and a few video-dependent predictors (e.g., man-made object importance, human-figure importance). Age and gender were related to curvature.

### Multi-Variable Relationships

As shown in Table 3, many of the potential predictor (fixed-factor) variables were related to saccade curvature and velocity-profile deviation in the two multi-variable models that included cubic polynomial fits, as shown. Many, but not all of the predictors found to be related to saccade curvature and velocity-profile deviation in the uni-variable models were included in the multi-variable models, and thus were independent predictors. Similarly, some factors that were significant in uni-variable models (e.g., scene cut during saccade) or even in the initial multi-variable model (e.g., importance of nature content), were not included in the final multi-variable model. Factors that were not included in the final model are shown with the significance from the full (all factors) model. Within the multi-variable models, the strongest effects were saccade-dependent variables (duration, orientation).

**Table 3:** Summary of the outcomes of the “multi-variable,” backwards-regression models of curvature and with velocity profile deviation.

	**Log-curvature**			**Log-velocity-profile deviation**		
	**coefficient**	***z*-value**	**significance**	**coefficient**	***z*-value**	**significance**
Log-Curvature				2.19	32.9	<0.001
Log-curvature^2^				1.03	22.3	<0.001
Log-curvature^3^				0.188	18.1	<0.001
Log-Velocity-profile deviation	2.28	38.7	<0.001			
Log-deviation^2^	0.971	40.8	<0.001			
Log-deviation^3^	0.142	44.8	<0.001			
Log-Duration	5.68	26.1	<0.001	21.6	64.5	<0.001
Log-duration^2^	−2.70	19.7	<0.001	−13.7	65.0	<0.001
Log-duration^3^	0.324	11.4	<0.001	3.00	68.7	<0.001
Vertical orientation^*^			0.45	0.0078	4.89	<0.001
Oblique orientation^*^	0.026	28.6	<0.001	−0.034	30.1	<0.001
Leftward saccade^*^	−0.0042	3.57	<0.001			0.69
Downward Saccade^*^	0.0059	5.44	<0.001	0.061	35.5	<0.001
Time-on-task			0.02			0.12
Inflight at scene cut			0.37			0.82
Number of cuts			0.52			<0.001
Environment (outdoor)			0.34	0.036	3.36	0.001
Nature-content importance			0.21			0.002
Face importance			0.04			0.07
Man-made object importance			0.29			0.26
Human figure importance	0.177	4.56	<0.001			0.09
Lighting			0.48	0.018	3.25	0.001
Auditory information			0.26			0.35
Movie genre^∇^	0.120	2.91	0.004			0.15
Age	0.00045	3.91	<0.001			0.05
Gender			0.86			0.23
Education			0.23			0.18

A positive coefficient indicates that the dependent variable (curvature or velocity profile deviation) increased with an increase in the value of the variable or increased in the presence of that variable (for binary variables). (^*^) Horizontal saccades were used as base level, so comparisons for vertical and oblique saccades are against horizontal saccades. Leftward saccades were compared to rightward saccades. Downward saccades were compared to upward saccades. (∇) Comedy had lower curvature and velocity-profile deviation compared to each of the other movie genres, documentary, animation, drama and action.

**Scene cut (shot change) during a saccade:** We predicted that when a scene cut occurred during a saccade, that saccade would have more curvature and greater velocity-profile deviations than saccades that did not have an in-flight scene cut. In the uni-variable analyses (Table 2) saccades with an in-flight scene cut had lower curvature (*p* < 0.001) and greater velocity-profile deviation (*p* < 0.001). However, those effects were not present when corrected for other variables (as reported in Table 3; multi-variable models).

**Saccade curvature:** was greatest when saccade duration was about 30 ms (or magnitude about 5 degrees; see Figure 5), and greater when saccades were oblique compared to horizontal (*p* < 0.001), and was greater when saccades were to the right (*p* < 0.001) or down (*p* < 0.001) than to the left or up, respectively (Table 3). Also, one video-dependent variable was significant, human-figure importance (*p* < 0.001), and curvature was greater with increasing age (subject-dependent factor) (*p* < 0.001; Table 3).

**Velocity-profile deviation:** was greatest when saccade duration (or magnitude) was longer (Figure 6). Velocity-profile deviation was lower with saccades that were vertical (*p* < 0.001) or oblique (*p* < 0.001) compared to horizontal and with saccades that were upward (*p* < 0.001) as compared to downward, respectively. Velocity-profile deviation was also related to some video-content factors, environment type (*p* = 0.001), and lighting (*p* = 0.001; Table 3). In the multi-variable model, we did not find significant effects in any of the subject-dependent factors.

Saccadic curvature and velocity-profile deviation were interrelated. As shown in Tables 2, 3, we modeled the velocity-profile deviation as a cubic polynomial of saccadic curvature (Figure 6) and saccadic curvature as a cubic polynomial of velocity-profile deviation. As shown in Figure 6, as curvature increases, velocity-profile deviation becomes slightly greater. Conversely, as velocity-profile deviation increased, curvature increased to about 0.07 units at about 0.0025 units of velocity-profile deviation, after which curvature was approximately stable with increasing velocity-profile deviation (not shown). Though the fits were significant (Tables 2, 3), the fits were not reversible and produced substantively different fits through the data, which reduces confidence in their meaning. This comes from the nature of linear regression in which the errors are minimized in the dimension of the predicted variable (plotted as the ordinate).

## Discussion

In our large sample of saccades made while watching movie and TV video clips, saccade curvature, velocity-profile deviation, and saccade duration (or magnitude) were inter-related. Also, saccade curvature and velocity-profile deviation varied strongly with saccade orientation. Interestingly, some aspects of the video content were related to curvature and velocity-profile deviation (as discussed individually below), including the lighting conditions, and human figures being important for understanding clip content, even when corrected for other factors (Table 3). Contrary to our hypothesis scene cuts were not related to saccade curvature or velocity-profile deviation, when corrected for other factors (Table 3).

Curved saccades were shown in very early studies (Lamasky, 1869; Yarbus, 1967) and can be elicited by elements or events in the scene, particularly when those occur during saccade planning or saccade flight. The mechanism for curved saccades may lie in the timing and relationships between motor-control pathways (McPeek et al., 2003; Arai and Keller, 2005). Saccade curvature has been mainly studied in the context of distracting, attracting and repelling objects that often have been made to occur during saccade flight. Given this, we hypothesized that the dynamic nature of movies might cause saccade curvature, since on-screen elements may change during a saccade and thus act as attractors or repellors, or the saccade target might move location during the saccade flight, triggering adjustments from the oculomotor control system. Deviation of saccade trajectory has been used as a measure of the oculomotor competition evoked by a distractor (McPeek and Keller, 2001). In our sample, saccades that were in flight during cut scenes, in the uni-variable models (Table 2), had lower saccade curvature (*p* < 0.001) and higher velocity-profile deviations (*p* < 0.001). However, in-flight scene cuts was not significantly related to either saccade curvature (*p* = 0.37) or velocity-profile deviations (*p* = 0.82), when corrected for other factors (multi-variable models; Table 3). Thus, scene cuts were not related to saccadic curvature or velocity profile deviation.

Some video-dependent factors were related to saccade curvature (Table 2). However, when corrected for other factors (e.g., saccade duration, orientation; Table 3), only the importance of human figures (e.g., people running) presented in the video clips was related to curvature, with lower curvature when human figures were important for understanding (*p* < 0.001). Perhaps, if human-figure stimuli were tracked during a saccade, their nature did not elicit large in-flight modifications of the saccade path (which would appear as higher curvature) or their movement could be predicted in saccade planning. The effect of distractors (stimuli that appeared during a saccade) on saccade curvature were greater when the distractors were salient (van Zoest et al., 2012) or had greater social relevance (Laidlaw et al., 2015), and varied with high-level (semantic) information related to the distractors (Weaver et al., 2011). Videos contain high-level information and salient and socially-relevant stimuli. The greater curvature with human-figure importance could indicate that the human figures had high salience. The importance of faces, man-made objects (e.g., cars) or nature were not related to the amount of curvature, even in the uni-variables analyses (Table 2), even though these were considered important for understanding of the video content. Curvature was lower with comedy videos than documentary (*p* = 0.004) or animations (*p* = 0.007; Table 3). It is possible that our documentary and animation clips had faster movements that could have induced curvature, but the clips labeled as action, did not have more curvature than the comedy clips (*p* = 0.15).

Similarly, in uni-variable analyses (Table 2), some video-dependent factors including when nature (*p* < 0.001), faces (*p* = 0.001), man-made objects (e.g., vehicles; *p* = 0.008) and human figures (*p* < 0.001) were related to velocity-profile deviations, but not when corrected for other factors (Table 3). Velocity-profile deviations were greater in outdoor than indoor scenes in the multi-variate analysis (*p* < 0.001), though, were not significant in the uni-variate analyses (Table 2).

A well-illuminated background enables the visual system to calculate target position faster and more accurately than when the target moves in the dark (Lemij and Collewijn, 1989). This is consistent with the finding that targeting saccades made by monkeys in dim background light had a shorter latency and were less hypometric than those in the dark (Straube et al., 1997). On that basis, we expected that curvature and velocity-profile deviation would be less when viewing brighter scenes. Contrary to that expectation, in the multi-variable models (thus corrected for other factors; Table 3), velocity-profile deviation was greater when the lighting conditions in the video clip were brighter (*p* = 0.001). Similarly, outdoor scenes, which were associated with higher lighting (*z* = 115, *p* < 0.001), was independently related to greater velocity-profile deviations (*p* < 0.001).

Wu et al. (2015) found that the number of shifts of the eyes to the center of the screen after presenting a scene change increased with the level of scene change, suggesting that the detection of a global context change may be required to trigger a re-evaluation of the scene. A higher number of cuts might be related to a greater number of saccades to the display center, and those might be more subject to curvature or velocity-profile deviation as they were not planned (driven by a new object of interest). In the uni-variable model, the number of cuts was not associated with curvature or velocity-profile deviation (Table 2), but was initially associated with velocity-profile deviation when all factors were included in the model (*p* < 0.001), but was not significant (*p* = 0.02) as other non-significant factors were removed (Table 3).

Di Stasi et al. (2013, 2014) found that time-on-task decreased saccadic velocity, presumably from fatigue. While fatigue impairs motor planning, our task of watching 10 to 30 min of video was not particularly fatiguing. Time-on-task was related to velocity-profile deviation (*p* < 0.001) in the uni-variable models (Table 2), but in the multivariable models (Table 3) time-on-task was not related to curvature (*p* = 0.02) or velocity-profile deviations (*p* = 0.12), when corrected for other factors.

Saccade curvature increased with increasing age (univariable and multi-variate models), which suggests that some aspect of the oculo-motor system is less able to control the eye movements. However, Pratt et al. (2006) found no differences in saccade kinematics between younger and older subjects.

Previously, we had noted that some saccades elicited while viewing movies had a velocity profile that was not well fit with the compressed exponential model (Han et al., 2013). That model accounts for the skewness of velocity profiles that has been studied previously (van Opstal and van Gisbergen, 1987; Takagi et al., 1993). We are aware of only one report of discrepancies in the velocity profile which was related to a cerebellar degeneration (Zivotofsky et al., 2006), though velocity-profile deviations are apparent in some previous publications. The lack of reports of velocity-profile deviations may be because such saccades are removed from off-line analyses because of their failure to comply with known models. When working with a real-time gaze-contingent system with saccade prediction, a response is required from the system for each saccade, whether the saccade looks normal or not. We had hypothesized that velocity-profile deviations might be related to curvature, with the discrepancies in the velocity profile being related to mid-flight changes in motor commands. Indeed, generally, velocity-profile deviations were larger when curvature was greater, but the relationship was not compelling, despite the high level of significance in our models, as the relationship was not strong, in the sense of large changes (Figure 6). Overall, longer saccades had greater velocity-profile deviation, and this was not because there was more opportunity for a discrepancy between the data and the model, since the root-mean-square normalizes for the amount of data (Equation 1).

Saccade curvature was greater for oblique than horizontal and vertical saccades, while velocity-profile deviation was greater for vertical than horizontal or oblique saccades (Table 3). We had hypothesized that horizontal saccades might be less subject to saccade curvature or velocity-profile deviation, there being fewer extraocular muscles involved in horizontal saccades, and thus less opportunity for errors in motor execution. This was based on models of the generation of saccades (Godijn and Theeuwes, 2002; Arai and Keller, 2005; Schreiber et al., 2006,) since there would be fewer opportunities for mistiming. We had hypothesized that mistiming might be a cause of difficult-to-predict saccades, however, our results are not consistent with that hypothesis. It is not clear to us, why rightward and downward saccades were related to greater curvature than rightward or upward saccades while downward saccades were associated with greater velocity-profile deviation (Table 3).

**Figure 8:**
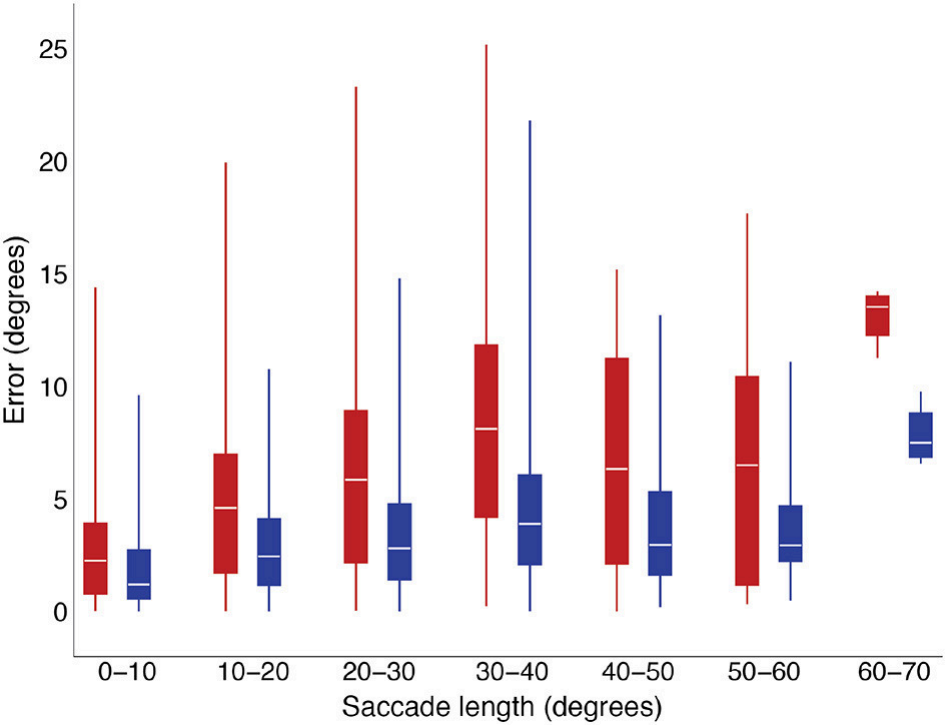
Gaze position deviations in a real-time, gaze-contingent system were smaller when using the Han et al. (2013) saccade-prediction algorithm (blue) than when not using saccade prediction (red).

Our purpose in conducting this exploratory study was to examine conditions under which saccade curvature or velocity-profile deviations occurred, as these features created discrepancies between the real-time prediction of eye position during a saccade as we do with our gaze-contingent system. System latency occurs even when using displays with high refresh rates (e.g., 100–120 Hz; Saunders et al., 2014). Previously, Han et al. (2013) showed that real-time saccade prediction reduces errors between location of a stimulus and eye position when calculated off-line. When that algorithm was implemented in our gaze-contingent system (i.e., runs in real time), there was a reduction in stimulus placement errors, as shown in Figure 8. That algorithm does not account for saccade curvature and assumes a conventional velocity profile (i.e., fit by compressed exponential function). If we knew when more-curved saccades or saccade with higher velocity-profile deviations were likely to occur, we could either plan an experiment to reduce the likelihood of occurrence (e.g., avoid conditions related to difficult-to-predict saccades, Table 3) or we could, in real-time, determine that there was a risk and implement a slower prediction method that has less prediction error. Recently, we have developed an algorithm that further reduces errors in prediction of eye position in the presence of saccade curvature and velocity-profile deviation (Wang et al., 2017). Interestingly, that algorithm is faster than the Han et al. (2013) algorithm and needs less data to start making a prediction, so has the potential to provide even better reduction of stimulus presentation location errors.

A major limitation of our study is that it was primarily observational, and therefore, cannot address the causes of curved saccades or causes of saccades with velocity-profile deviations. Another limitation is that we used an infra-red-video-based eye tracking system. Though the data is not presented here, we have observed similar curvature and velocity profile deviations in data collected using a dual-Purkinje-image system (Han et al., 2013) and a scleral-coil system (Costela et al., 2014, 2015; Wang et al., 2017), which suggests that the effects reported here are not an artifact of the video-based eye-tracking system (Hooge et al., 2016). These results suggest that distractors are not essential to elicit saccades with strong curvature and velocity-profile deviations. In future studies, we will compare and analyze these saccadic features during the performance of different visual tasks.

## Author Contributions

FC and RW: Designed the experiment, analyzed the data, and wrote and revised the manuscript. FC: Collected the data.

### Conflict of Interest Statement

The authors declare that the research was conducted in the absence of any commercial or financial relationships that could be construed as a potential conflict of interest.
