# Supplementary material for: When Watching Video, Many Saccades Are Curved and Deviate From a Velocity Profile Model
Source: Front Neurosci. 2019 Jan 7;12:960. doi: 10.3389/fnins.2018.00960 (PMC6330331; doi:10.3389/fnins.2018.00960)
Supplement: Supplementary file 1 [file Presentation_1.pdf]

Supplement to Costela & Woods (2018)

Alternative processing of the data set was conducted using the Eye-Markup analysis tool (Berg et al., 2009a). The Eye-Markup detection algorithm applied the same initial velocity threshold as our algorithm (30 deg/sec) to detect saccadic intervals but is used in combination with a simple windowed Principal Components Analysis (PCA). The PCA window filter classifies each time sample as an eye-movement event (fixations, saccades, blinks, smooth pursuits and blends of smooth pursuit, optokinetic, and saccadic eye movements events) using a substantial number of thresholds and multiple iterative 'cleaning' sequences to refine events within the eye gaze. Consequently, we found that the Eye-Markup algorithm was very slow and used substantial memory such that the dataset had to be processed in subsets. Here, we present an analysis of the data collected while subject viewed the 30-second video clips (so, about half of the total data set).

Before processing, the Eye-Markup algorithm uses a 63 Hz low-pass Butterworth filter for data smoothing. Initially, we used a similar low-pass filter, but found that it caused a few problems, most importantly being that it caused a small-time displacement. Thus, for our saccade-detection algorithm, we used a 3rd-order Savinsky-Sgolay filter with a frame size of 15.

We did not explore this systematically, but it seems that the Eye-Markup finds slightly different start and end points (in time or space) to those found by our saccade-detection algorithm. Thus, the two algorithms will find that the saccade magnitude or duration of the same saccade to be different. The difference might be a fixed number of data points, in which case the proportional difference will vary between the algorithms. This has implications for the saccade main sequence, as the maximum velocity will be the same, but the saccades may shift in a non-linear manner along the saccade magnitude axis. For saccade curvature, since the point-wise curvature method includes all data points in the identified saccade, if the start and end points differ, and curvature is high at one end of the saccade (e.g. Figure 2b), it is possible that the curvature metric will vary slightly between the two algorithms for the same saccade.

Overall, the results using the Eye-Markup algorithm were broadly comparable to the results found using our saccade-detection algorithm and the full data set, as described in Table 3 of the primary paper (Costela and Woods, 2018). Curvature and velocity-profile deviation values remained significantly dependent on each other, along with duration/length and orientation factors (see table S1). The main sequence of the saccades detected by the Eye-Markup algorithm (Figure S1A) seems to have a broader upper band (compare to (Costela and Woods, 2018); Figure 1A). Also, it identified a smaller proportion of vertical saccades. Thus, we considered the Eye-Markup detection algorithm less robust than our speed-threshold algorithm when used on our human, video-watching data set. Another possible explanation for this discrepancy may be the fact that the Eye-Markup analysis tool was tuned for the analysis of eye movements in a monkey dataset (see (Berg et al., 2009b).

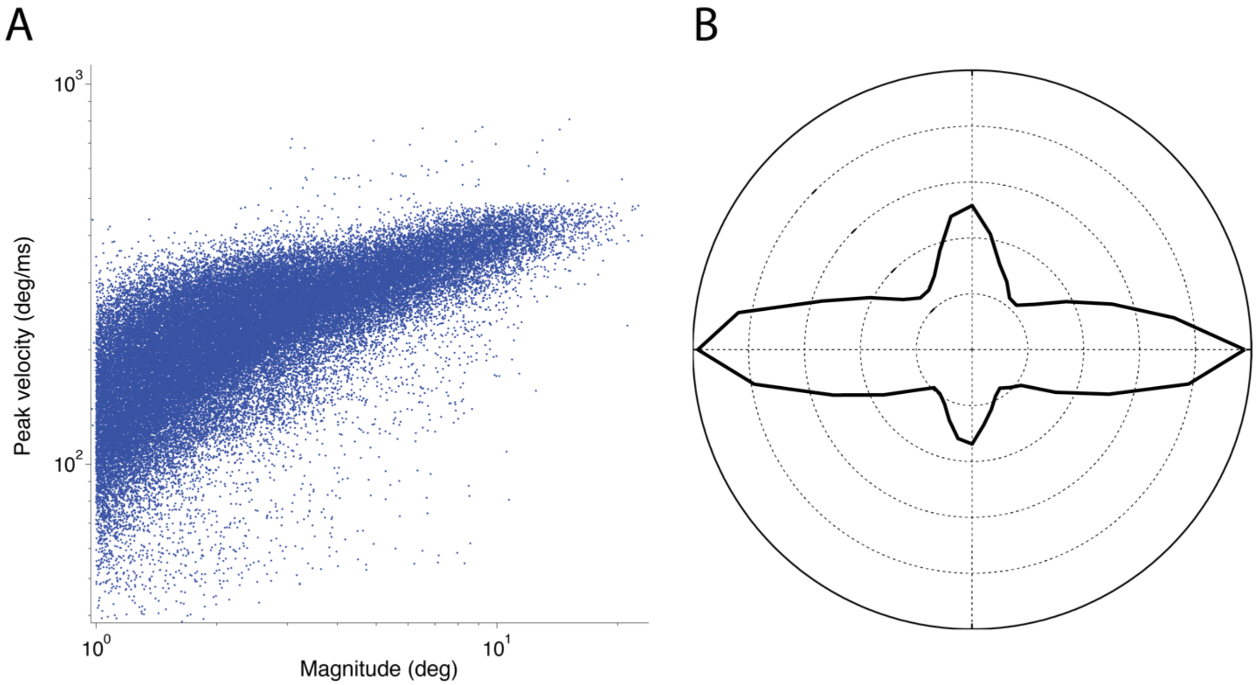

Figure S1. Saccadic features for 61 subjects and 108,153 saccades using the Eye-Markup saccade detection algorithm. A) Saccadic peak velocity–magnitude main sequence. The distribution is plotted on logarithmic scale where peak velocity is indicated on the y-axis and magnitude indicated on the x-axis. B) Frequency distribution of saccade directions in polar co-ordinate orientations.

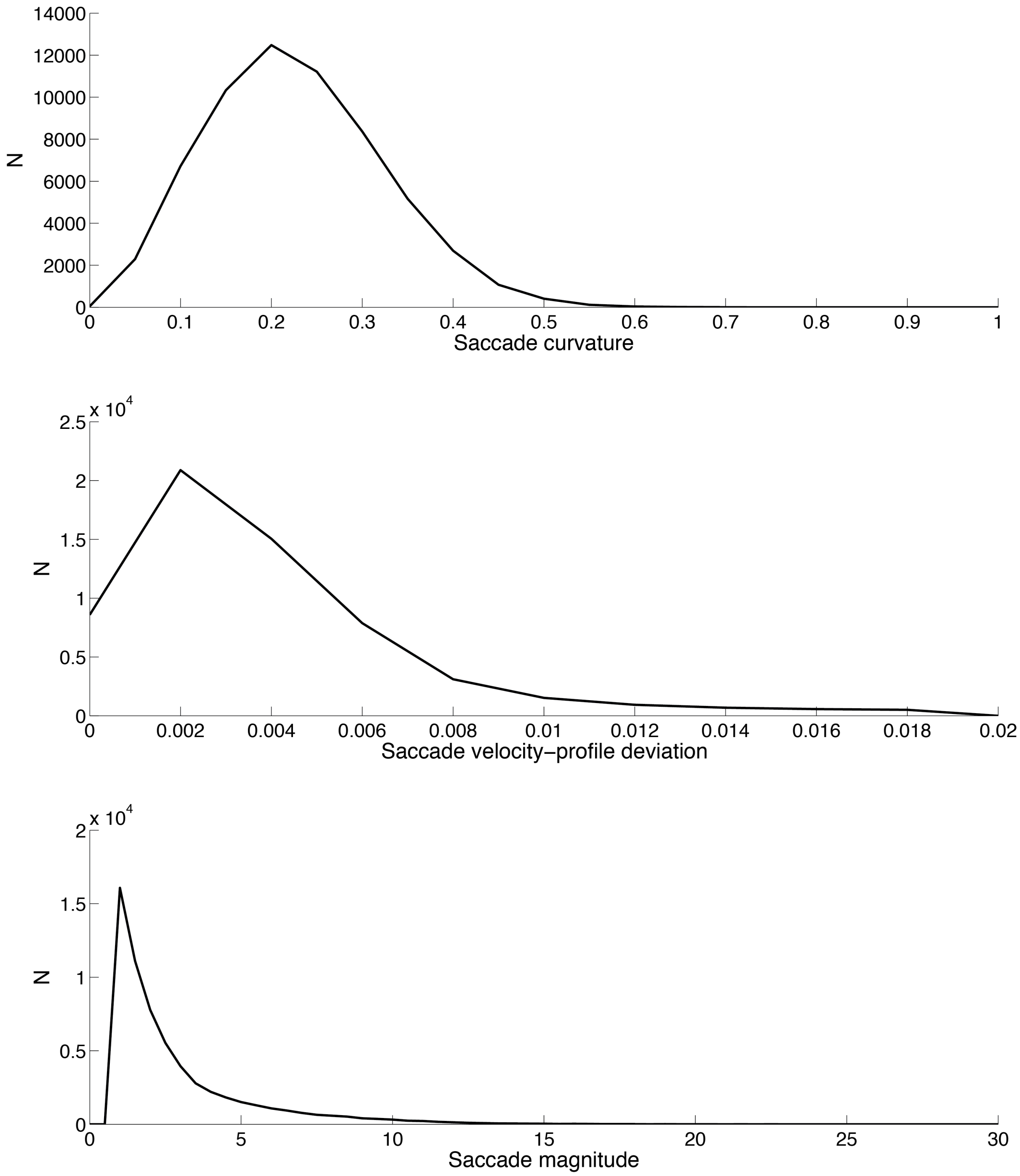

Figure S2. Saccade features using the Eye-Markup saccade detection algorithm. Both curvature and velocity-profile deviation distributions shared the same shapes as compared to the original detection algorithm, although shifted, given the different criteria of the detection algorithm. In particular, the Eye-Markup algorithm differed from our detection algorithm in the use of different filters - They used a low-pass Butterworth cutoff filter to smooth the gaze, while we used a Savinsky-Sgolay filter. Additionally, they added a series of thresholds to classify each time sample as an eye-movement event (fixations, saccades, blinks, smooth pursuits and blink-saccade events) by using a PCA window filter to refine intervals around saccades. Consequently, these thresholds may account for the larger curvature and velocity-profile deviation values that we found in the dataset when using our saccade-detection algorithm (A) Saccade curvature ranged from 0.037 to 1.326 units (median: 0.349). (B) Saccade velocity-profile deviation ranged between 0.0003 and 0.084 deg/s (median: 0.015). (C) Saccade magnitude ranged between 1 and 35.8 deg (median: 3.973 deg, compared to median 4.1 deg by our detection algorithm).

Table S1. Summary of the outcomes of "multi-variable", backwards-regression models of curvature and with velocity profile deviation of saccades identified using the Eye-Markup saccade detection algorithm. A positive coefficient indicates that the dependent variable (curvature or velocity profile deviation) increased with an increase in the value of the variable or increased in the presence of that variable (for binary variables). Horizontal saccades were compared to not-horizontal saccades and likewise for vertical and oblique saccades. Leftward saccades are compared to rightward saccades. Downward saccades are compared to upward saccades.

	Log-Curvature			Log-Velocity-profile deviation		
	coefficient	z-value	significance	coefficient	z-value	significance
Log-Curvature				0.160	4.39	<0.001
Log-curvature^2				0.696	10.6	<0.001
Log-curvature^3				0.213	5.77	<0.001
Log-Velocity-profile deviation	0.751	7.43	<0.001			
Log-deviation^2	0.411	8.19	<0.001			
Log-deviation^3	0.142	6.53	<0.001			
Log-Duration	0.0534	7.21	<0.001	-12.6	20.3	<0.001
Log-duration^2	-2.88	9.71	<0.001	7.46	19.3	<0.001
Log-duration^3	0.713	11.7	<0.001	-1.45	18.1	<0.001
Vertical orientation*	0.0352	21.1	<0.001	0.00806	4.28	<0.001
Oblique orientation*	0.0196	14.6	<0.001	0.0147	11.0	<0.001
Leftward saccade*	0.0080	4.66	<0.001			0.56
Downward Saccade*	0.0059	5.44	<0.001	0.0443	20.5	<0.001
inflight at scene cut			0.06	-0.0230	2.61	0.009
Number of cuts			0.14			0.02
Environment (outdoor)			0.52			0.75
Nature-content importance			0.04			0.07
Face importance			0.08			0.45
Man-made object importance			0.65			0.09
Human figure importance			0.02			0.19
Lighting			0.34			0.88
Auditory information			0.38			0.88
Age	0.00087	4.42	<0.001	0.000917	2.77	0.006
Male	0.0345	5.19	<0.001	0.114	9.90	<0.001
Education			0.23	-0.0141	3.67	<0.001

References

Berg, D., Shen, J., and Itti, L. (2009a). The eyeMarkup eye movement analysis tool. . Retrieved from http://ilab.usc.edu/cgi-bin/secure/viewcvs.cgi/trunk/saliency/matlab/Eye-Markup/.

Berg, D.J., Boehnke, S.E., Marino, R.A., Munoz, D.P., and Itti, L. (2009b). Free viewing of dynamic stimuli by humans and monkeys. *Journal of Vision* 9**,** 5, 19 11-15.

Costela, F.M., and Woods, R.L. (2018). When watching video, many saccades are curved and deviate from a velocity profile model. *Frontiers in Neuroscience* 12**,** 960.
